# Brain changes due to hypoxia during light anaesthesia can be prevented by deepening anaesthesia; a study in rats

**DOI:** 10.1371/journal.pone.0193062

**Published:** 2018-02-16

**Authors:** Setayesh R. Tasbihgou, Mina Netkova, Alain F. Kalmar, Janine Doorduin, Michel M. R. F. Struys, Regien G. Schoemaker, Anthony R. Absalom

**Affiliations:** 1 Department of Anaesthesiology, University Medical Centre Groningen, University of Groningen, Groningen, the Netherlands; 2 Department of Nuclear Medicine and Molecular Imaging, University of Groningen, Groningen, the Netherlands; 3 Department of Anaesthesia, Ghent University, Gent, Belgium; 4 Department of Molecular Neurobiology, GELIFES, University of Groningen, Groningen, the Netherlands; University of Modena and Reggio Emilia, ITALY

## Abstract

In anaesthetic practice the risk of cerebral ischemic/hypoxic damage is thought to be attenuated by deep anaesthesia. The rationale is that deeper anaesthesia reduces cerebral oxygen demand more than light anaesthesia, thereby increasing the tolerance to ischemia or hypoxia. However, evidence to support this is scarce. We thus investigated the influence of light versus deep anaesthesia on the responses of rat brains to a period of hypoxia. In the first experiment we exposed adult male Wistar rats to deep or light propofol anaesthesia and then performed [^18^F]- Fludeoxyglucose (FDG) Positron Emission Tomography (PET) scans to verify the extent of cerebral metabolic suppression. In subsequent experiments, rats were subjected to light/deep propofol anaesthesia and then exposed to a period of hypoxia or ongoing normoxia (n = 9–11 per group). A further 5 rats, not exposed to anaesthesia or hypoxia, served as controls. Four days later a Novel Object Recognition (NOR) test was performed to assess mood and cognition. After another 4 days, the animals were sacrificed for later immunohistochemical analyses of neurogenesis/neuroplasticity (Doublecortin; DCX), Brain Derived Neurotrophic Factor (BDNF) expression and neuroinflammation (Ionized calcium-binding adaptor protein-1; Iba-1) in hippocampal and piriform cortex slices. The hippocampi of rats subjected to hypoxia during light anaesthesia showed lower DCX positivity, and therefore lower neurogenesis, but higher BDNF levels and microglia hyper-ramification. Exploration was reduced, but no significant effect on NOR was observed. In the piriform cortex, higher DCX positivity was observed, associated with neuroplasticity. All these effects were attenuated by deep anaesthesia. Deepening anaesthesia attenuated the brain changes associated with hypoxia. Hypoxia during light anaesthesia had a prolonged effect on the brain, but no impairment in cognitive function was observed. Although reduced hippocampal neurogenesis may be considered unfavourable, higher BDNF expression, associated with microglia hyper-ramification may suggest activation of repair mechanisms. Increased neuroplasticity observed in the piriform cortex supports this, and might reflect a prolonged state of alertness rather than damage.

## Introduction

The aim of anaesthesia is to render the patient unconscious, and thus insensible to the pain and suffering of surgery, and thereafter ensure a safe and complication free recovery.

Different anaesthetists apply different strategies in an attempt to optimize safety and to optimize the quality of recovery from anaesthesia and surgery. Some aim for lighter anaesthesia, by administering lower doses of the anaesthetic drugs, since lighter doses are associated with fewer hemodynamic adverse effects, such as hypotension, and a more rapid recovery of consciousness once drug administration is stopped. Furthermore, some anaesthetists might opt for lighter anaesthesia because of recent evidence that the anaesthetic agents have neurotoxic effects (particularly in the brains of the very young and the elderly) and because of controversial evidence suggesting that deeper anaesthesia is associated with a worse 1 year mortality rate [1–3]. Light anaesthesia however is associated with a risk of inadvertent return of consciousness during supposed anaesthesia, which is a feared complication of anaesthesia [4]. To avoid this problem of ‘awareness’ anaesthetists commonly administer deeper anaesthesia. Many anaesthetists consider that anaesthetic exposure has no long-term consequences for the brain, and indeed there is evidence that anaesthesia might be neuroprotective [3]. Systemic hypotension is common during surgery, and may cause cerebral ischemia and/or hypoxia, which in turn may be associated with neuronal damage and impaired postoperative cognitive outcome [5,6]. Most of the currently used anaesthetic agents, such as propofol and isoflurane are Type A γ-Aminobutyric acid (GABA_A)_ agonists, which potentiate γ-Aminobutyric acid(GABA)-mediated inhibition of synaptic transmission and thereby cause dose-dependent suppression of cerebral metabolism [5–11]. By inhibiting cerebral metabolism, these agents reduce cerebral oxygen and glucose demand, and hence may prolong the time the brain can withstand ischemia or hypoxia, without major neuronal damage. Accordingly, when cerebral ischemia or hypoxia are anticipated, anaesthetists will administer deep anaesthesia to protect the brain [12,13]. However, evidence to support this practice is scarce. In the present study, we thus investigated the influence of light versus deep anaesthesia on the responses of rat brains to a period of hypoxia. We hypothesized that exposure to hypoxia during light anaesthesia would cause changes in the brain, and that deep anaesthesia would attenuate these changes.

## Materials and methods

### Animals and ethical approval

Male Wistar rats (weight range 380 – 500g) were purchased from Harlan (Horst, The Netherlands) and housed for 2 weeks before the start of the experiments in the local animal facility (Centrale Dienst Proefdieren, Groningen, the Netherlands) under controlled conditions with a 12/12-hour dark/light cycle with ad libitum access to food and water. The study was approved by the local animal experiment and welfare committee (Dier Experimenten Commissie, Groningen, the Netherlands, DEC6281), and performed in accordance with the submitted protocol and local procedural regulations.

Using the Knibbe pharmacokinetic model for propofol we performed mathematical simulations, to design two propofol infusion schemes, aiming to achieve light or deep propofol anaesthesia [14]. Two separate studies were then performed: study 1 to verify the extent of metabolic suppression when deep or light propofol anaesthesia was administered using these schemes (n = 20) and study 2 to investigate effects of hypoxia or normoxia in the presence of deep or light anaesthesia (n = 46).

### Experimental protocol

#### Study 1

After induction of anaesthesia by isoflurane, rats (n = 20) were randomized to receive either high or low dose propofol via a catheter inserted in the tail or penile vein, to achieve deep or light anaesthesia respectively. Once target concentrations were reached and the animals appeared to be in a stable plane of anaesthesia for at least two minutes, they underwent Positron Emission Tomography (PET) scanning to obtain measurements of brain metabolism.

#### Study 2

After induction of anaesthesia with isoflurane, rats (n = 41) were randomized to receive either light or deep propofol anaesthesia. After 15 minutes of propofol anaesthesia, rats were subjected to either 10 minutes of hypoxia or remained normoxic, resulting in 4 experimental groups; light anaesthesia-normoxia (n = 11); light anaesthesia-hypoxia (n = 11); deep anaesthesia-normoxia(n = 9); deep anaesthesia-hypoxia (n = 10). Subsequently, all rats were ventilated at normoxia for another 30 minutes, allowed to awaken and then transferred back to their cage. Naïve rats, not exposed to anaesthesia (and without hypoxia) served as controls (n = 5). Four days later, a Novel Object Recognition (NOR) test was performed to assess mood and cognitive function. It is known that surgery can have long lasting effects on the rats’ ability of recognizing objects, as such this decision was made in order to assess the rats after 4 days in order to demonstrate the long-term effects of hypoxia during anaesthesia on cognitive decline [15]. On day 8, rats were sacrificed, brains were collected and processed for immunohistochemical analysis of neurogenesis, neuronal functioning and neuroinflammation.

### Experimental procedures

#### Induction and maintenance of anaesthesia

Anaesthesia was induced by placing the rat into a clear plastic box containing 2–3% isoflurane in a 50–50% mixture of O_2_ and air. In rats that underwent PET scanning (study 1), after induction, the trachea was intubated and the lungs mechanically ventilated (Amsterdam Infant Ventilator; HoekLoos) at a rate of 50/min using the same mixture as during induction. Tidal volume was set to achieve normocapnia verified by capnography and arterial blood gas analysis. The remaining rats (study 2) undergoing anaesthesia, received a 50–50% mixture of O_2_ and air administered via a face mask, with spontaneous ventilation.

The tail or penile vein was cannulated and a bolus dose of propofol (40mg/kg or 160mg/kg in the light and deep anaesthesia group, respectively) was administered. After exactly 2 minutes, propofol infusion was administered using a target controlled infusion scheme, designed based on the Knibbe model to reach 2 μg/ml (light anaesthesia) and 8 μg/ml (deep anaesthesia) effect site concentration, respectively [14]. Heart rate and oxygenation were monitored with a pulse oximeter. The tail artery was cannulated (26G catheter) for blood pressure monitoring and arterial blood gas analysis. If hypotension was noticed, dopamine was administered to maintain Mean Arterial Pressure (MAP) between 60–80 mmHg. The physiological parameters measured can be found in Supporting Information as Appendix (S1 Appendix). The body temperature of the rats was kept at 37.5±0.5°C using an electrical heating pad.

After at least two minutes of anaesthesia, the animals underwent the PET scanning procedure or the hypoxia/normoxia intervention.

#### [^18^F] Fludeoxyglucose (FDG) Positron emission tomography scanning

[^18^F]–FDG PET scans were performed to verify extent of cerebral metabolic suppression during deep versus light anaesthesia (n = 10 per group). The rats were positioned into the small animal PET scanner (Focus 220, Siemens Medical Solutions, USA, Inc.) in a transaxial position with their heads in the field of view. A transmission scan of 515 seconds with a ^57^Co point source was obtained for the correction of attenuation and scatter by tissue. After the transmission scan was completed, [^18^F] FDG was injected intravenously (injected dose 19±3 MBq). Simultaneously with the injection of [^18^F] FDG, an emission scan of 60 min was started. The list-mode data of the emission scans was separated into 21-frame sinograms (8x30, 2x60, 2x120, 2x150, 3x300 and 3x600 seconds), which were iteratively reconstructed (OSEM2D, 4 iterations, 16 subsets) after being normalized and corrected for attenuation, scatter, randoms and decay. PET image analysis was performed using *VINCI* 4.12 (Max Planck Institute for Neurological Research Cologne, Germany) More detail on the approach can be found in the protocol in S1 Appendix.

#### Hypoxia and normoxia

The rats breathed spontaneously via a breathing mask, through which a hypoxic or a normoxic mixture was administered for 10 minutes. In the normoxic group, room air (21% O_2_) was administered. In the hypoxic group, a 5–95% mixture of O_2_ and N_2_ was initially administered via a standard rotameter bank. The administered oxygen fraction was then adjusted until the peripheral capillary oxygen saturation (SpO_2_) was between 50–55%.

After 10 minutes of hypoxia/normoxia all rats were ventilated with 21% O_2_ for another 30 minutes, allowed to spontaneously awaken and then transferred back to their cage in the animal facility.

#### Novel Object Recognition (NOR) test

On day 4 after anaesthesia and hypoxia induction the NOR test was performed to assess working memory function that relies primarily on the rats’ innate exploratory behaviour [16]. The NOR procedure was performed as follows: each rat received a training phase of ten minutes, and one hour later, a testing phase of three minutes. During the training phase, rats were placed in a box with dimensions 65 x 45 x 45cm (height) with two identical objects (two light bulbs) and allowed to explore the area. After ten minutes, the rats were placed back into their cage. All objects were cleaned with 70% ethanol before they were used in order to remove smell cues. One hour later, the rats were placed back in the box, with one object exchanged for a novel one (a jar). After three minutes, each rat was put back in the cage. The training phase and testing phase were both recorded with a camera and analysed at a later time point (*DOSBox 0*.*74* programme for behavioural data acquisition, *Eline* Version 0.91 (Groningen)). The percentage of time the rats spent exploring each object during the training and testing phase was measured and scored objectively in a blinded manner and in a random order rats. The time spent exploring the 2 identical objects was taken as a measure of “interest in environment”, as reduced interest in the environment can be associated with negative affective states such as depression or anxiety. The ratio of time the animal spent exploring the novel object expressed as percentage of the total object exploration was taken as a measure of novel object recognition.

#### Sacrifice and harvesting of organs

On day 8, the animals were sacrificed by transcardial perfusion with saline containing 0.1% EDTA, under brief deep isoflurane anaesthesia. The brains were removed, rinsed, and immersion fixed in 4% paraformaldehyde for 4 days followed by cryoprotection with 30% sucrose in Phosphate-Buffered Saline (PBS) and then frozen at -80ºC for immunohistochemical analysis.

### Immunohistochemical analysis

The brains were sectioned with a cryostat *Leica CM 3050* into 25 μm thick sections, and free floating sections were further pretreated with 3% H_2_O_2_ for 20 min. After staining, the sections were mounted for image analysis. Images of the Doublecortin (DCX) and the ionized calcium-binding adaptor protein-1 (Iba-1) stained sections were acquired with an *Olympus BH2* microscope with a *Leica DFC320 CCD* camera using an appropriate software (Leica Microsystems, The Netherlands). The image analysis for the stainings was performed using *Image-Pro Plus* 6.0.0.26 (Media Cybernetics, Inc.) The Brain Derived Neurotrophic Factor (BDNF) stainings were analysed directly using the Leica software. The analyses were done by observers blinded to the experimental groups in three sections per animal.

#### Doublecortin (DCX)

Brain slices were stained for DCX to visualize adult neurogenesis in the Dentate Gyrus (DG). DCX is a protein that is expressed in a precise manner in migrating neuroblasts during early embryonic development, as well as in neurogenic areas in the adult brain. In addition, DCX positive cells in the piriform cortex, a sensory area adjacent to the PET-scan indicated affected area, were analysed as they may reflect neuroplasticity [17]. As control, DCX positivity was obtained in the basolateral amygdala by measuring optical density. The detailed protocol for the staining can be found in S1 Appendix. A quantitative dimension of DCX positive cells was obtained by dividing the area of DCX positive cells by the length of chosen DG region [17].

#### Ionized calcium-binding adaptor protein-1 (Iba-1)

To visualize microglia, we used the ionized calcium-binding adaptor protein-1 (Iba-1), which is an actin-binding protein that is specifically expressed in microglia and is used as an immunohistochemical marker for both ramified and activated microglia [18]. The detailed protocol for the staining can be found in S1 Appendix. Microglia morphology was used as parameter for neuroinflammation and was analysed as described in detail by Hovens et al [19]. For that, the number of microglia per region, the average total cell size, and average total cell body size (expressed as pixels per high power field) were determined at 200x magnification. Subsequently, average dendrite area was calculated. Microglia activation is expressed as cell body per cell size ratio, and is indicated as a relevant marker for neuroinflammation [19]. Microglia analysis was obtained in the radial layer of the Dentate Gyrus-inner blade (DGib), Cornu Ammonis 1 (CA1) and Cornu Ammonis 3 (CA3) region of the hippocampus. To check for area specificity, microglia were also analysed in the piriform cortex (layer containing DCX positive cells).

#### Brain derived neurotrophic factor (BDNF)

Based on our findings regarding neuroinflammation and neurogenesis, we also aimed to investigate step in between to further elucidate the underlying mechanism of the brain changes. Therefore, in the most relevant experimental groups; controls (n = 5), hypoxia-light anaesthesia (n = 8), and hypoxia-deep anaesthesia (n = 9), we performed an additional staining on BDNF in the hippocampus. Detailed description of the protocol can be found in the S1 Appendix.

The optical density of each of three reference areas were measured for each hippocampal section. The reference areas were: CA1 region, CA3, and DGib. Each reference area was compared to the background optical density in real-time, high-resolution imaging using the Leica Application Suite (LAS) Macro. However, since the staining was not uniform throughout the sections, to maintain consistency the optical density of the reference area was measured against a local background area in the same region.

### Statistical analysis

For the Iba-1, DCX, BDNF and NOR analysis, SPSS (IBM SPSS Statistics for Windows, 20.0.0.2) was used to calculate the mean and the Standard Error of Mean (SEM), and to perform statistical analysis. We analysed the multiple experimental group means by two-way analysis of variance (ANOVA) with the factors "anaesthetic depth (An)" and "oxygenation (Ox)", followed by a Bonferroni post-hoc analysis. P-values <0.05 were considered significant. Furthermore, correlations were assessed using Pearson’s correlation. Correlations with P-values <0.05 were considered significant.

Regarding the PET-scans, all data are expressed as mean ± standard deviation and further analysed by one-way ANOVA, with significance when the p-value <0.05. Statistical analysis was performed using SPSS (IBM SPSS Statistics for Windows, version 18.0).

## Results

### General

In study 1, a reliable PET scan could be obtained from only 17 of the 20 rats (n = 9 for light anaesthesia, n = 8 for deep anaesthesia). In study 2, 41 rats were subjected to anaesthesia, 6 of which could not complete the study due to death during anaesthesia or technical problems, resulting in the following group sizes: normoxia-light anaesthesia n = 9; hypoxia-light anaesthesia n = 8; normoxia-deep anaesthesia n = 8; and hypoxia-deep anaesthesia n = 10. The control group not exposed to anaesthesia or hypoxia consisted of 5 rats.

### [^18^F] FDG small animal positron emission tomography scan

**Fig 1:**
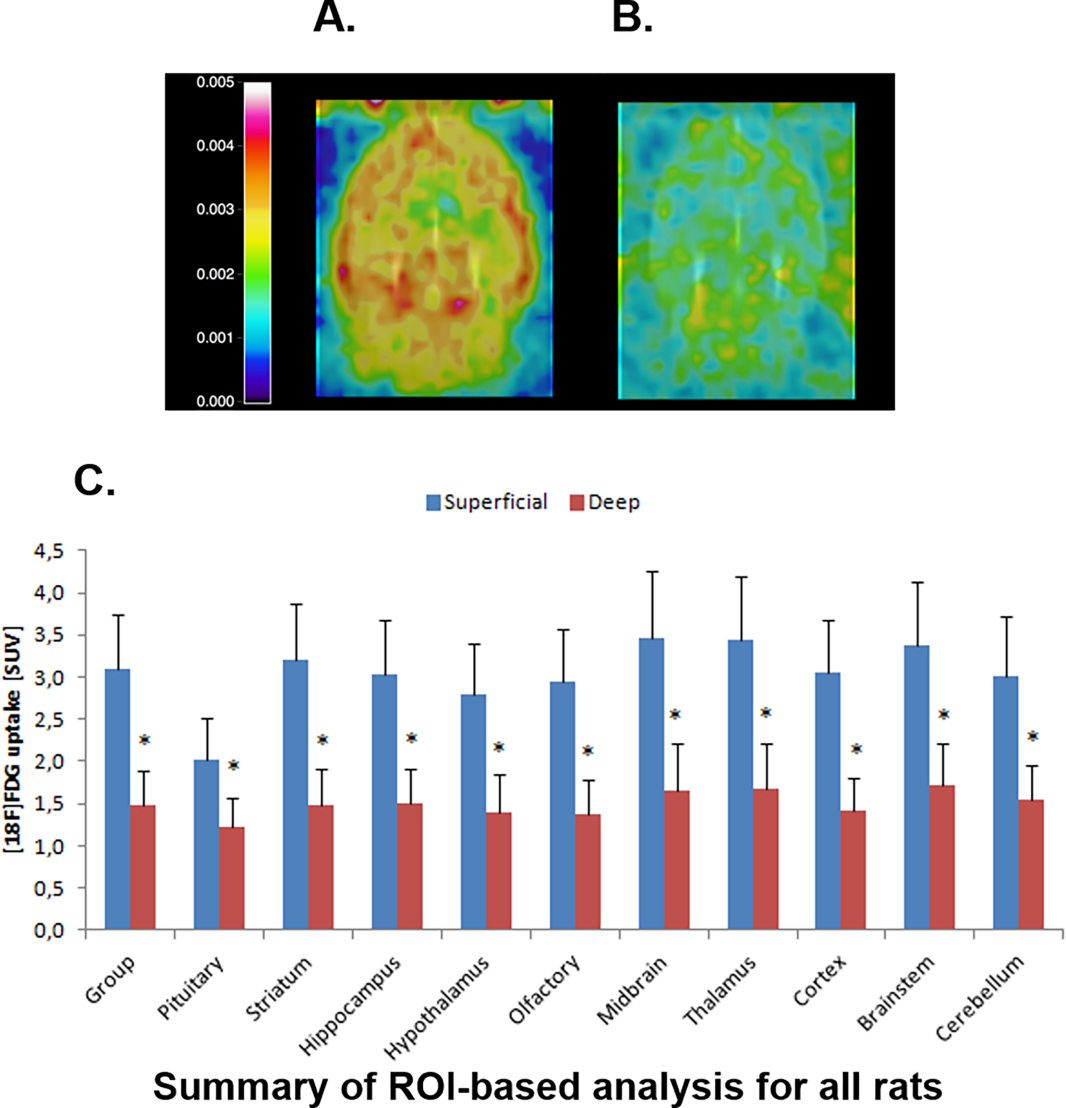
[^18^F] FDG Positron Emission Tomography (PET) scan results. [^18^F] FDG PET scan co-registered with an MRI template of a rat under light propofol anaesthesia (A), and deep propofol anaesthesia (B). Fig 1C shows the results of the ROI-based analysis depicting the mean [^18^F] FDG uptake across all animals subjected to light (superficial) and deep propofol anaesthesia. Data were analysed by one-way ANOVA. *: significant difference between regions.

In Fig 1, representative examples of [^18^F]-FDG PET scan images from a rat that underwent light propofol anaesthesia (Fig 1A), and a rat that underwent deep anaesthesia (Fig 1B), are depicted. These show lower [^18^F]-FDG uptake across the whole brain during deep than during light anaesthesia. This observation is substantiated by the region-of-interest (ROI) based analysis (Fig 1C) showing that [^18^F]-FDG uptake was significantly lower for all brain regions in the rats undergoing deep anaesthesia than those undergoing light anaesthesia (39–53% lower, p<0.005 for all regions).

### Novel object recognition (NOR) test

**Fig 2:**
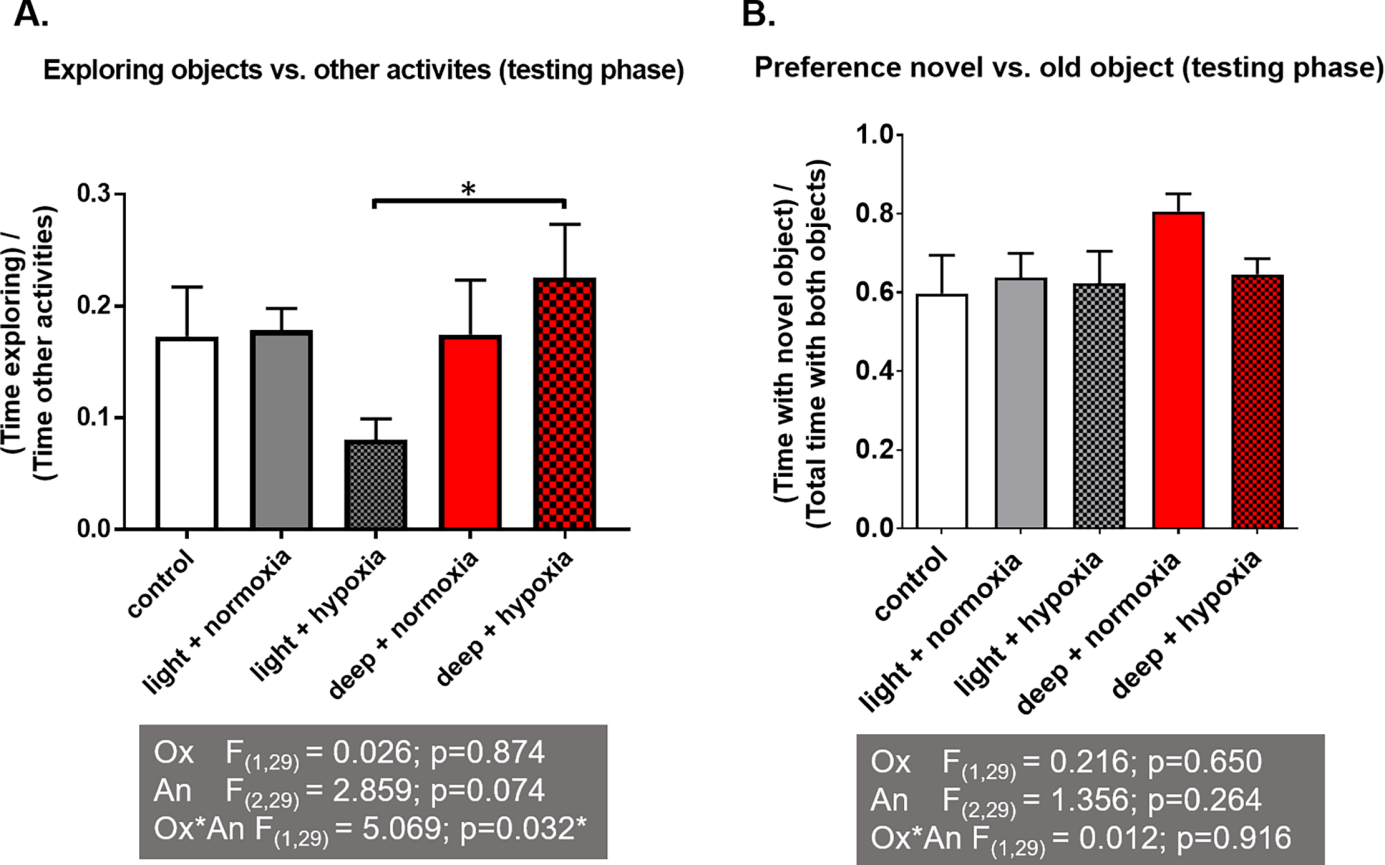
Novel Object Recognition (NOR) test 4 days after anaesthesia. Mean time spent exploring the objects vs. performing other activities (A) and preference over the novel object vs. the old object (B) during the testing phase of the NOR test. The multiple experimental group means were analysed by two-way ANOVA with the factors "anaesthetic depth" (An) and "oxygenation" (Ox) followed by a Bonferroni post-hoc analysis. *: significant difference between groups.

Cognitive performance was measured with the novel object recognition test. In the NOR test analysis, data of 35 rats were available for analysis. During the training phase with two identical objects, the rats did not show preference for the left or right object (5.6±0.5 and 5.6±0.8%, respectively). There was reduced exploration of both objects in the hypoxia-light anaesthesia group compared with the other groups, including a significant difference with the hypoxia-deep anaesthesia group (Fig 2A). Moreover, the rats of the hypoxia-light anaesthesia group spent significantly more time immobile. In the testing phase, all groups showed preference for the novel object (significantly different from change level), without differences between the groups (Fig 2B).

### Immunohistochemical brain analysis

#### Doublecortin (DCX) staining

**Fig 3:**
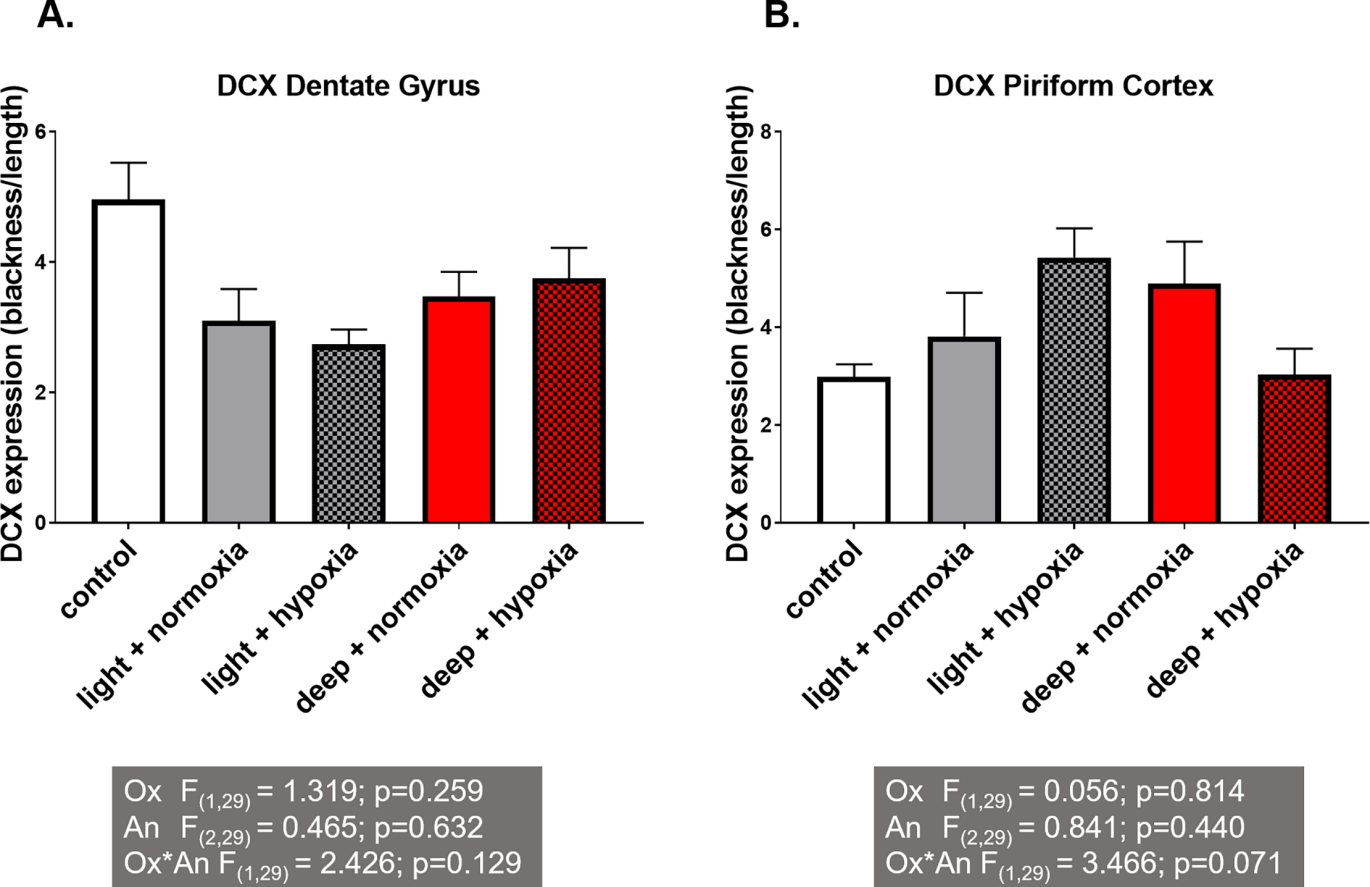
Doublecortin (DCX) immunohistochemical brain analyses. Mean ± Standard Error of Mean DCX expression found in the Dentate Gyrus (A) and Piriform cortex (B). The multiple experimental group means were analysed by two-way ANOVA with the factors "anaesthetic depth" (An) and "oxygenation" (Ox) followed by a Bonferroni post-hoc analysis. *: significant difference between groups.

DCX staining was analyzed in three different regions of the brain: the dentate gyrus, the piriform cortex and the lateral amygdala area. In the dentate gyrus of the hippocampus there was a 45% reduction in the number of DCX-positive cells in hypoxia-light anaesthesia group compared to the control group; the reduction in the hypoxia-deep anaesthesia group was minimal (Fig 3A). Likewise, in the piriform cortex, hypoxia-light anaesthesia rats displayed a minimal increase in DCX positivity compared to the control, an effect that was reduced to control levels by deep anaesthesia (Fig 3B). No difference in DCX expression between the experimental groups was observed in the lateral amygdala.

#### Ionized calcium-binding adaptor protein-1 (Iba-1) staining

**Fig 4:**
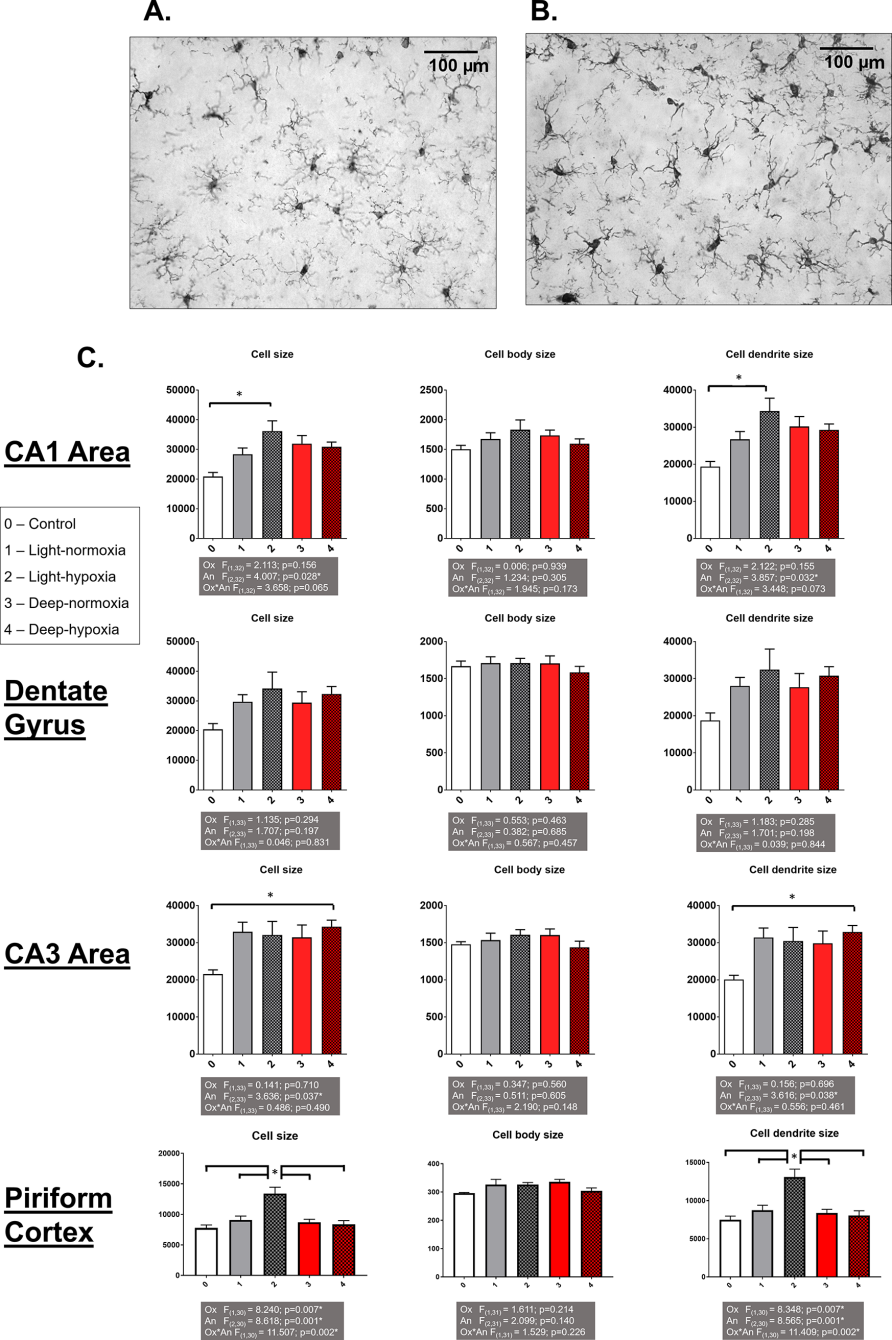
Ionized calcium-binding adaptor protein-1 (Iba-1) immunohistochemical analysis of the hippocampus. An example of Iba-1 stained microglia images obtained from the CA1 region of the hippocampus with a 200x magnification showing control group stains (A) and hypoxia-light anaesthesia stains (B) and overview per region of hippocampus (CA1, Dentate Gyrus, CA3) and Piriform Cortex of the mean ± Standard Error of Mean total cell size of microglia (first column), cell body size (second column) and cell dendrite size (third column) (C). The multiple experimental group means were analysed by two-way ANOVA with the factors "anaesthetic depth" (An) and "oxygenation" (Ox) followed by a Bonferroni post-hoc analysis. *: significant difference between groups.

In Fig 4, examples of Iba-1 stained microglia images in the CA1 region of the hippocampus are depicted for a control rat (Fig 4A) and a light anaesthesia-hypoxia rat (Fig 4B). The photographs display microglia hyper-ramification while the cell body size is unchanged after administering hypoxia during light anaesthesia. This observation is substantiated with actual measurements as presented in Fig 4C.

The number of microglia cells did not differ significantly among any of the groups for all hippocampal areas (CA1: 14.47±0.44; DG: 15.54±0.61; CA3: 15.45±0.63 per high power field). Microglia activation is characterized by an increased cell body size and shortening of the dendritic processes. In the CA1 area, total microglia cell size is increased in the hypoxia-light anaesthesia group compared to control, which is not present anymore when anaesthesia is deepened. This can be attributed to a significant increase in dendrite area, as cell body area is not changed. Similar effects, though not statistically significant, can be observed in the DG area, but not in the CA3 area. In the latter, all anaesthesia groups, independent of concomitant oxygen levels, displayed an increased cell size, attributable to increased dendrite area. This reached statistical significance only in the hypoxia-deep anaesthesia group. (Fig 4C).

In the piriform cortex, the number of microglia per high power field was significantly lower in the hypoxia-light anaesthesia group compared to all other groups (control: 20±1; normoxia-light anaeshtesia:19±1; hypoxia light anaesthesia: 15±1; normoxia-deep anaesthesia: 20±1; hypoxia-deep anaesthesia: 21±1). This is accompanied by a significantly increased cell size that could be attributed to increased dendrite size (Fig 4C).

#### Brain derived neurotrophic factor (BDNF) staining

**Fig 5:**
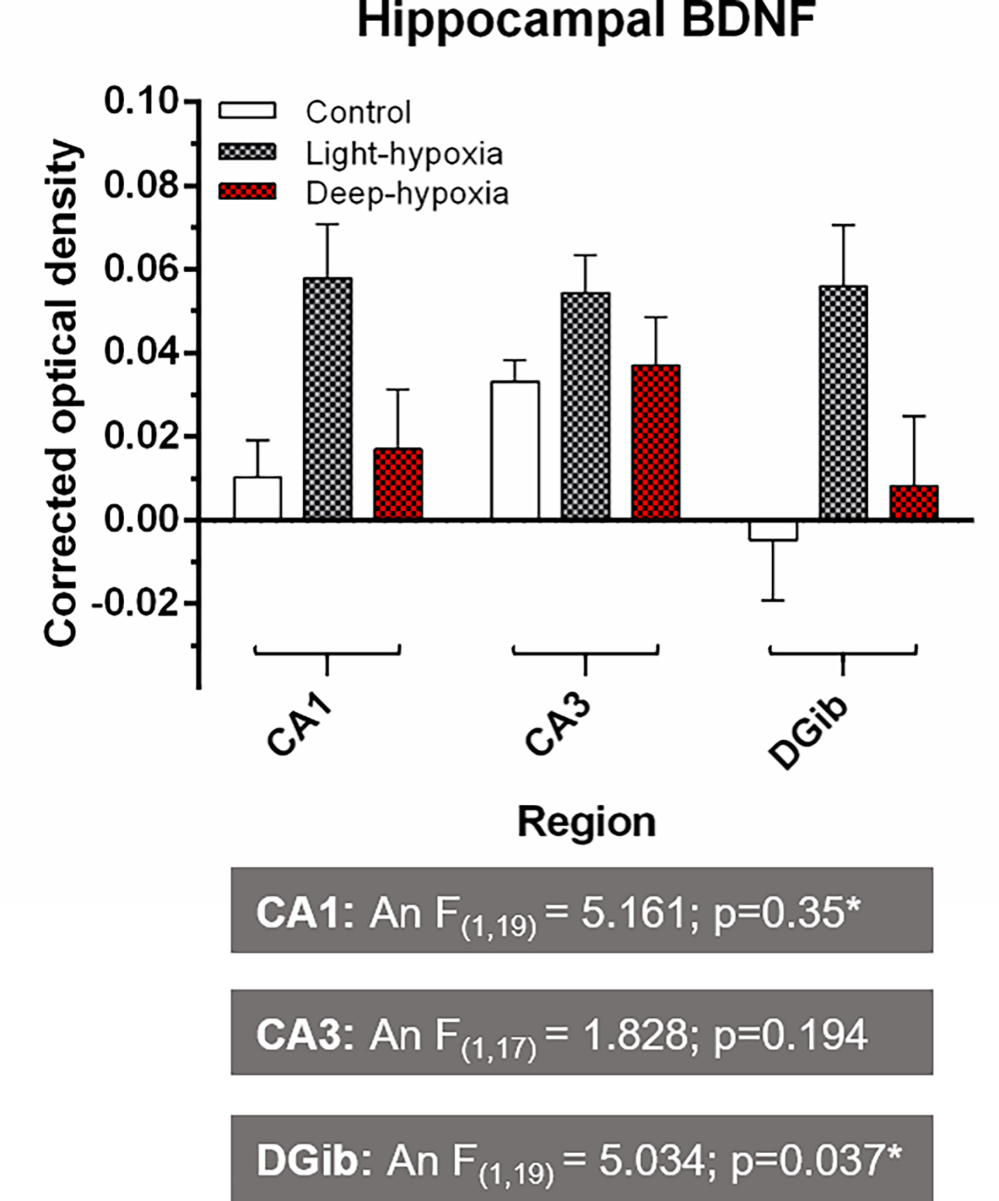
Immunohistochemical analysis of Brain Derived Neurotrophic Factor (BDNF) in the hippocampus. Optical density measurements of BDNF staining in the hippocampus for control, light anaesthesia–hypoxia and deep anaesthesia -hypoxia animals. CA1: Cornu Ammonis 1; CA3: Cornu Ammonis 3; DG: Dentate Gyrus inner blade. The multiple experimental group means were analysed by two-way ANOVA with the factors "anaesthetic depth" (An) and "oxygenation" (Ox) followed by a Bonferroni post-hoc analysis. *: significant difference between groups.

Fig 5 presents the BDNF staining in the hippocampus. The corrected optical density for the CA1, CA3 and DG are shown for the control, hypoxia-light anaesthesia and hypoxia-deep anaesthesia rats. ANOVA depicted significant differences between groups for CA1 and DG, but post-hoc analysis only showed significant differences between control and hypoxia-light anaesthesia in the DG.

### Correlations

**Fig 6:**
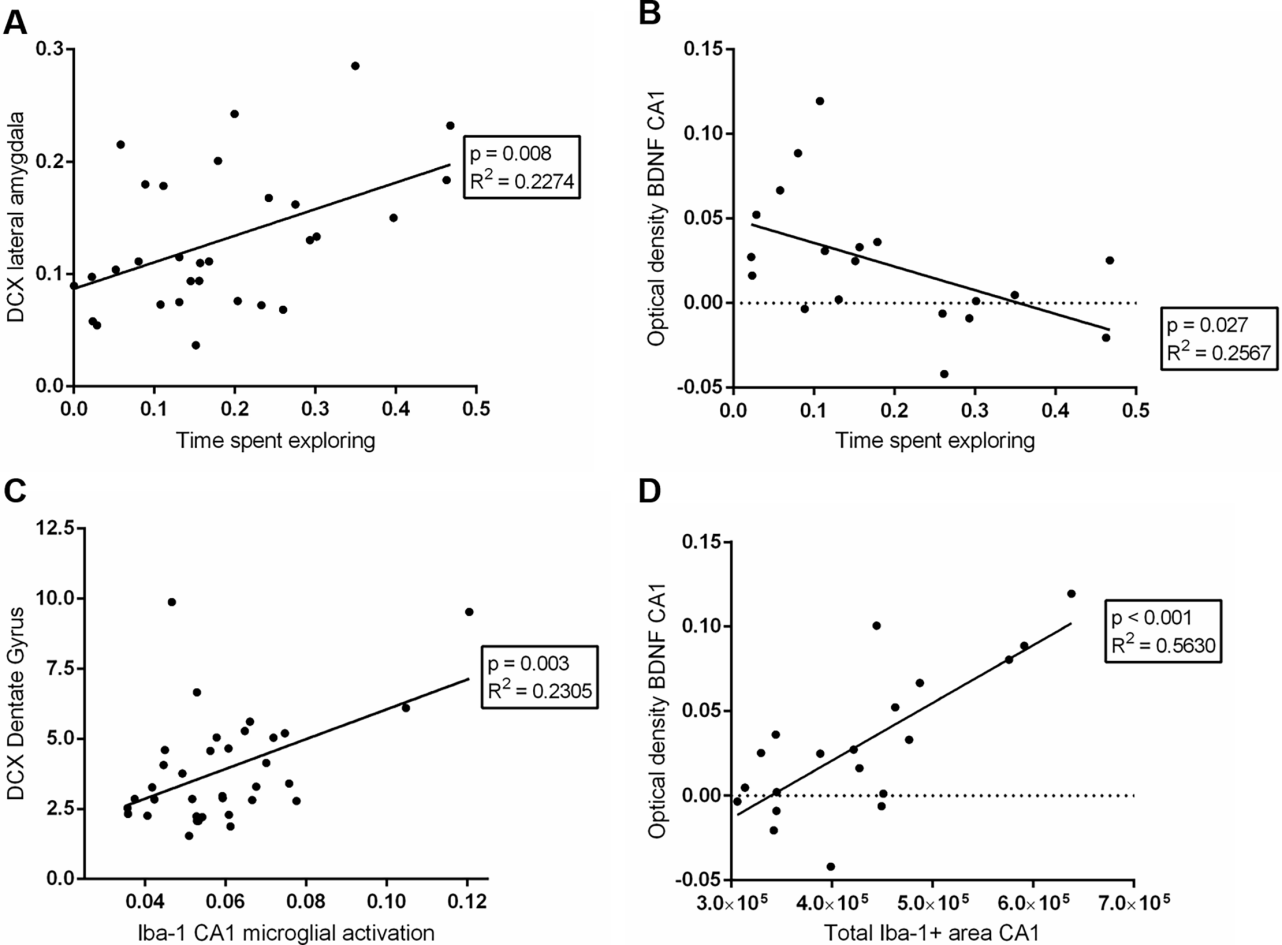
Illustrations of various correlations between immunohistochemical and cognitive tests. Time spent exploring was positively correlated with the expression of DCX in the lateral amygdala (A). The presence of BDNF in the Cornu Ammonis 1 (CA1) was negatively correlated the time spent exploring (B). There was a positive correlation between DCX in the dentate gyrus and Iba-1 microglial activation as calculated for the CA1 (C). BDNF expression in CA1 was also positively correlated with the total Iba-1 positive area for CA1 (D).

Correlations between relevant parameters were analysed. Firstly, cognitive performance, measured by the preference for the new object in the NOR test, was not different between groups and was not correlated to any obtained parameter. However, time spent on exploration of the objects is significantly positively correlated with DCX staining in the amygdala (Fig 6A), and negatively to microglia cell body size and BDNF staining (Fig 6B) in the CA1 and DG region. Moreover, it appeared negatively correlated with microglia dendrites in the piriform cortex. Neurogenesis, measured as DCX staining in the DG, is not found to be correlated to microglia parameters in the DG, but it is significantly correlated to microglia activity (Fig 6C), cell size and dendrite area in the CA1 area. Furthermore, a significant positive correlation was found for BDNF and total measured positive Iba-1 area for CA1 (Fig 6D) and DG. Similarly, a correlation was found between BDNF and Iba-1 positive cell size and dendrite area for CA1, but not for CA3 or DG. Finally, a significant correlation was observed between piriform cortex DCX and microglia dendrites in this area (p = 0.002). Tables for each correlation can be found in the S1 Appendix.

## Discussion

In this study, we aimed to investigate whether the depth of anaesthesia at the time of a hypoxic episode would affect the brain, which could be associated with changes in cognitive function. We hypothesized that exposure to hypoxia during light anaesthesia would cause changes in the brain, and that deep anaesthesia would attenuate these changes. We had anticipated that hypoxia during light anaesthesia would cause alterations in the brain because of the mismatch between cerebral oxygen demand and supply. Deeper anaesthesia, which reduces metabolism and hence oxygen demand might thus attenuate these changes. Indeed, hypoxia during light anaesthesia caused significant changes in the brain and behaviour. The observed hippocampal reduction in neurogenesis (DCX) was associated with decreased exploration rather than cognitive dysfunction. However, neurogenesis was positively correlated with microglia activity in the hippocampus, suggesting activation of compensation (neuroinflammatory) mechanisms rather than deterioration. Hippocampal microglia hyper-ramification, concomitant with increased BDNF, as well as higher neuroplasticity (DCX) observed, correlated with microglia hyper-ramification in the piriform cortex would support this. The above effects are lower or absent in the hypoxia-deep anaesthesia group, indicating that indeed deep anaesthesia may prevent changes in the brain afflicted by a mismatch between oxygen demand and oxygen supply during hypoxia-light anaesthesia.

The target organ of the anaesthetic agents is the brain, so anaesthetic depth is best quantified on the basis of the effects on the brain. With increasing depth of anaesthesia within clinical ranges, most anaesthetic agents increasingly suppress cerebral electrical activity as detected by the Electroencephalogram (EEG) [20] and cause a dose-dependent depression of the cerebral metabolic rate [3,5–7,9,10,21] up to a nadir of about 40% of baseline (awake) metabolic rate [20]. Accordingly, the PET scans confirmed that the magnitude of the glucose uptake (i.e. metabolic rate) during deep anaesthesia was almost half of that under light anaesthesia–suggesting that the selected doses were indeed appropriate for light and deep anaesthesia. Glucose and oxygen consumption are likely to change roughly in parallel, indicating a reduced oxygen demand at the higher dose of propofol administered in the present study.

### Anaesthetic effects on behaviour and cognition

In clinical studies, Postoperative Cognitive Dysfunction (POCD), comprising impairment of memory, attention and executive functions, has been strongly linked to anaesthesia and surgery and particularly to intraoperative ischemia (with resultant cerebral hypoxia) [1,22,23]. In our experiments, rats who were exposed to hypoxia and received light anaesthesia spent significantly less time (compensated for by immobility) exploring the objects, but did not display lower interest in the novel object. Rats showing less interest in their environment could be interpreted as signs of anxiety and depression, and/or a response to sickness or illness [22,24,25], which would be supported by the correlation we found with changes in the amygdala, an area involved in affective behaviour. This finding supports the hypothesis that hypoxia during low dose anaesthesia is associated with changes in the brain, and that deep anaesthesia attenuates this. However, preference for the novel object, as a measure of cognition (working memory) is preserved in all experimental groups, indicating no cognitive decline in this respect due to the anaesthesia regimes used. Additionally, our findings show that rats under deep anaesthesia show no changes in behaviour regardless of hypoxia exposure, suggesting that deep anaesthesia preserves this aspect of cognitive function and has no negative effects by itself.

### Anaesthetic effects on neurogenesis/neuroplasticity

Doublecortin (DCX) is a cytoskeleton-associated protein that is transiently expressed during adult neurogenesis and neuroplasticity [17]. It is associated with neuronal migration, axonal guidance and dendrite sprouting. The specific function of DCX in adult hippocampal neurogenesis is unknown, but in most instances, it can be used as a reliable measure for neurogenesis. DCX positive cells are present in adult rodent brains, including but not limited to the dentate gyrus of the hippocampus, piriform cortex, corpus callosum, and the lateral ventricle or amygdala [17]. Interestingly, in our study, we found different changes for different brain regions. In the hypoxia-light anaesthesia rats the DCX positivity was reduced by 45% in the DG of the hippocampus, whereas in the piriform cortex DCX expression was almost doubled. Deeper anaesthesia in both regions reversed this effect towards control values. Hippocampal DCX expression may represent neurogenesis, while piriform cortex DCX expression may relate to neuroplasticity [17]. As we were able to show a significant positive correlation between hippocampal microglial activity and hippocampal neurogenesis, increased microglia activity here may be regarded as an attempt to compensate for the reduced neurogenesis. Alternatively, microglia activation and neurogenesis may follow different time courses after anaesthesia [22]. Studies have found that the olfactory bulb, functionally related to the piriform cortex, is vulnerable to anaesthetics, showing neuro-apoptosis throughout the lifetime of a mouse [26]. In the piriform cortex the increased DCX expression may rather reflect neuroplasticity [17] and is concomitant with strong microglia hyper-ramification, indicating a cytoprotective action [17]. If anything, for all groups of anaesthetised rats the mean DCX expression in the lateral amygdala was equally reduced in comparison to controls. The lateral amygdala is thought to be responsible for fear learning and aversive behaviour including aggression and anxiety, which is suggesting that the lateral amygdala may be sensitive to the anaesthetic agents, rather than to hypoxia [22,27]. However, a highly significant correlation between amygdala DCX and object exploration would support its contribution to the observed effects in hypoxic-light anaesthesia conditions.

### Anaesthetic effects on neuroinflammation

Iba-1 allows visualization of brain microglia [19]. This protein, which is constitutively expressed in all microglia, is used to identify the functional state of microglia [19]. Compared to quiescent ramified microglia, microglia in the activated amoeboid state exhibit increased Iba-1 expression. In response to injury, morphological changes in classically activated microglia include shortened dendrites with simultaneously increased cell body size [28,29]. However, alternatively activated microglia show opposite morphological changes and are associated with a regulatory role in synaptic activity [30,31]. These hyper-ramified microglia are associated with non-pathological stimuli, such as stress, and may relate to depressive behaviour [30]. In our study, we observed a significant increase in total microglial cell size. This increase could be attributed to increased dendrite area of microglia cells, as cell body area remained unchanged. This finding shows that the microglia do not convert into the classically pro-inflammatory activated phenotype associated with short dendrite length and larger cell body size. Rather than distinct phenotypes, recently a wide spectrum of changes has been proposed for these supporting cells with a strong overlap in gene expression and functional portfolios [28,32,33].

Although additional staining for inflammatory markers would need to be performed to confirm the inflammatory state of the microglia, the Iba-1 morphological changes in rats from the hypoxia-light anaesthesia group may reflect a form of microglia priming It is well known that microglia can be “sensitized” or primed for prolonged periods of time [28]. This is often the result of prolonged or previous inflammatory stimulation [28,32,33]. These microglia are preconditioned to become hyperactive once activated. Priming of microglia has been found to be caused by chronic mild inflammation, prion disease, complement signalling and aging [28]. As exposure to anaesthesia, particularly to inhalational anaesthesia, has been purported to cause an acceleration of neurodegenerative processes, it is likely that the combination of light anaesthesia and hypoxia induced oxidative stress can result in microglial changes in the rats [3,28,34–36]. A property of primed microglia is that they fail to resolve from their hypersensitive state, and will not respond to anti-inflammatory cytokines. It has been speculated that microglial hyperactivation gives rise to depression-like behaviour and cognitive deficits [30]. This suggestion would be supported by the negative correlation between microglia dendrites in the piriform cortex and exploration behaviour (depressive-like) in the present study.

### Brain derived neurotrophic factor (BDNF) staining

Inflammation can directly and indirectly influence key regulatory functions of neurons through factors such as neurotrophins. One such neurotrophin is BDNF [37,38]. Traditionally, BDNF has been used as a marker for activation of intracellular cascades that regulate neuroplasticity, synaptogenesis and neurogenesis [22,39–43]. Furthermore, BDNF is linked with essential executive cognitive functions such as memory [44,45]. Alternatively, precursors to BDNF have been associated with growth factor induced neuro-apoptosis under some conditions. Previous studies have shown that neuro-inflammation decreases BDNF expression [37,40,46–48]. Interestingly, our findings showed that BDNF expression was markedly increased in animals from the hypoxia-light anaesthesia group. Furthermore, there was a positive correlation between Iba-1 expression (and dendrite area) and BDNF expression in the CA1 of the hippocampus, indicating that neuro-inflammation and microglia hyper-ramification was associated with an increased BDNF expression. This finding may be explained by the antibodies used for BDNF staining, which do not differentiate between BDNF and its precursors. Mature-BDNF (mBDNF) supports survival of neurons through Tropomyosin Receptor Kinase B (TrkB receptors), whereas pro-BDNF, the precursor to mBDNF, is involved in synaptogenesis, but can also induce neuronal apoptosis through p75-Neurotrophic Receptors (p75NTR) [46,47]. Anaesthetics have been shown to enhance p75NTR signalling, and thus to promote BDNF dependent neuroapoptosis in the brain[37,46–48]. This phenomenon may have occurred in all our anaesthetized rats, but the combination of light anaesthesia and hypoxia would then significantly have increased BDNF-dependent apoptosis. An alternative explanation for the increased BDNF in the hypoxia-light anaesthesia group is that the BDNF expression occurred because of a compensatory release of neurotrophic factors by hyper-ramified microglia, in response to reduced neurogenesis (as measured by DCX). It has been proposed that reduced neurogenesis can stimulate mechanisms that increase the expression of BDNF, and BDNF pathways for neuroplasticity and neurogenesis, for instance upregulation of TrkB receptors[22].

### Limitations

Some limitations of our study should be mentioned. Firstly, the effects were studied in rats, who only underwent anaesthesia. In clinical settings, anaesthesia is almost always administered to facilitate a surgical procedure. The surgical insult generates an inflammatory event and can activate or exacerbate systemic inflammation [22,49]. However, this study did provide an opportunity to study effects of different anaesthesia regimes, without the interference of effects of surgery. Secondly, we chose one time point to measure the effects of a process that likely will encompass a time course lasting weeks. Additionally, cognitive function as the ultimate outcome of expected neuronal damage was investigated in only one test, merely testing working memory. As all rats performed well, this test may not be sensitive enough to distinguish between the different interventions. It is unknown if the changes identified in the rat brain tissue after 8 days are longer-lasting or reversible. Future studies should include measurements to determine the long-term effects of (light and deep) anaesthesia and hypoxia. Thirdly our findings are based on a relatively limited sample size, selected pragmatically as there were no available satisfactory data to conduct an a-priori sample size calculation. A fourth limitation is that with the current methodology isoflurane was administered prior to propofol administration and again prior to sacrifice. So-called anaesthetic pre-conditioning, which can be stimulated by isoflurane might therefore have pre-activated defence and repair systems and thus may have influenced the outcome [50].

## Conclusion

Our data indicate that hypoxia during light anaesthesia is associated with significant immunohistological evidence of neuronal changes in comparison with other combined conditions. Although reduced hippocampal neurogenesis may indicate unfavourable effects, higher BDNF correlated to microglia hyper-ramification may suggest compensation for this effect. This is further supported by increased neuroplasticity in the piriform cortex. As deep anaesthesia during hypoxia at least partly attenuated all effects, we conclude that the effects of hypoxia during light anaesthesia may indeed be prevented by deep anaesthesia. Further research is required to confirm whether these effects occur in other pre-clinical settings, with larger sample sizes, and can be extended to the surgical arena and translated into clinical settings.

## Supporting information

S1 Appendix Supporting information on methods and staining. Detailed overview of protocols used for Positron Emission Tomography and the various immunohistochemical analyses with corresponding photographs of each staining and tables for physiological parameters measured during the experiment and the results for all correlations performed in this manuscript.
(DOCX)
