# Correction: Brain changes due to hypoxia during light anaesthesia can be prevented by deepening anaesthesia; a study in rats

**DOI:** 10.1371/journal.pone.0303455

**Published:** 2024-05-03

**Authors:** Setayesh R. Tasbihgou, Mina Netkova, Alain F. Kalmar, Janine Doorduin, Michel M. R. F. Struys, Regien G. Schoemaker, Anthony R. Absalom

In Fig 5, the ANOVA test result values for the CA1 region of the hippocampus are incorrect. It should be F(1,19) = 5.318; p = 0.033*. Please see the correct Fig 5 here.

**Fig 5:**
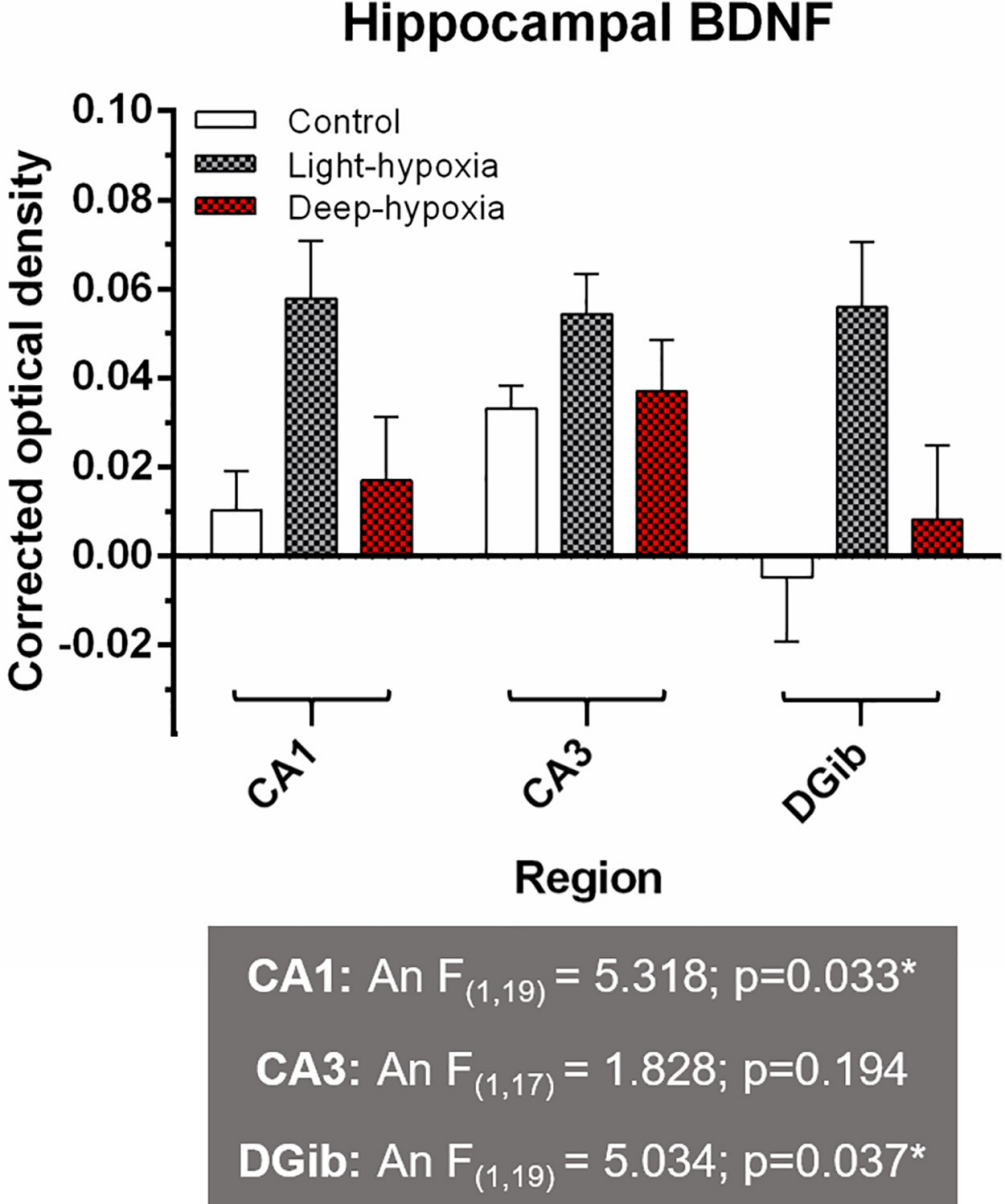
Immunohistochemical analysis of Brain Derived Neurotrophic Factor (BDNF) in the hippocampus. Optical density measurements of BDNF staining in the hippocampus for control, light anaesthesia–hypoxia and deep anaesthesia -hypoxia animals. CA1: Cornu Ammonis 1; CA3: Cornu Ammonis 3; DG: Dentate Gyrus inner blade. The multiple experimental group means were analysed by two-way ANOVA with the factors "anaesthetic depth" (An) and "oxygenation" (Ox) followed by a Bonferroni post-hoc analysis. *: significant difference between groups.
