# Supplementary material for: Brain changes due to hypoxia during light anaesthesia can be prevented by deepening anaesthesia; a study in rats
Source: PLoS One. 2018 Feb 16;13(2):e0193062. doi: 10.1371/journal.pone.0193062 (PMC5815614; doi:10.1371/journal.pone.0193062)
Supplement: S1 Appendix — Detailed overview of protocols used for Positron Emission Tomography and the various immunohistochemical analyses with corresponding photographs of each staining and tables for physiological parameters measured during the experiment and the results for all correlations performed in this manuscript. (DOCX) [file pone.0193062.s001.docx]

## S1 Appendix – supporting information

## Iba-1 protocol

The coronal sections were on day 1 incubated with rabbit anti-Iba1 (1:2500, Wako chemicals, Neuss, Germany) in 1% bovine serum albumin (BSA) in phosphate-buffered saline (PBS) containing 0.1% TritonX for 72 hours on 4ºC. On day 4, after 3 washes with PBS, the sections were incubated with biotin conjugated goat-anti rabbit secondary antibody (1:500, Jackson, Wet Groove, USA) for 2 hours at room temperature. After 6 washes with PBS, the sections were incubated with avidin-biotin peroxidase complex (1:500, Vectastain ABCkit, Vector, Burlingame, USA) for 1 hour at room temperature. After rinsing 3 times, the sections were stained with a tablet of DAB (15ml) solved in 30 ml water and 100 μl of 0.1% hydrogen peroxide. The reaction was stopped after 8 minutes. After a final 3 times wash with PBS, the sections were transferred to glass slides in a 1% gelatin solution and dehydrated through gradients of ethanol and xylol solutions.

## DCX protocol

After slicing the brain, the slices were rinsed twice with 0.01 M PBS. Then they were incubated with 3% hydrogen peroxide for 20 minutes and after that rinsed with PBS for four times. Subsequently, the slices were incubated with 5% normal rabbit serum in PBS for one hour. Next, the slices were incubated with AB Doublecortin (c-18) goat Santa Cruz at 37 °C for 3 hours, after which they were kept at a room temperature overnight and finally stored in a cold room for 3 nights. After this they were rinsed 5 times with PBS and then incubated with the second antibody (AB rabbit-anti goat Jackson), and kept in a cold room overnight. Then they were rinsed with PBS 5 times and incubated with ABC at room temperature for two hours. After being incubated the slices were again rinsed with PBS 3 times, kept at room temperature for two hours and then in a cold room overnight. Finally, they were incubated in a DAB solution which was activated with 0.1% hydrogen peroxide in Milli-Q. When the reaction was finished the slices were rinsed with PBS three times and prepared to be analyzed.

## PET scan image analysis protocol

[^18^F]-FDG uptake was found to be stable after about 25 minutes post-injection and uptake from 25-60 minutes post-injection was chosen for comparison between groups. Therefore frame 18-21 were summed to obtain a single frame image. Of these single frame images voxel-wise parametric standardized uptake value (SUV) images were constructed using the following formula: [tissue activity concentration (Bq/cm3)]/[injected dose (Bq)/body weight (g)]. It was assumed that 1 cm3 of brain tissue equals 1 g. Following the construction of SUV images, all images were aligned with a stereotactic MRI template using VINCI 4.12 and its automatic registration plugin [1]. New images were resliced with cubic voxels of a size of 0.2 mm size, and then scaled by a factor of 10 to resemble approximately the human brain size.

ROI-based analysis was performed to determine the effect of depth of anaesthesia on [^18^F]-FDG uptake in the whole brain and ten pre-defined brain regions. Using an MRI template (in VINCI 4.12), ROIs were drawn around the whole brain, olfactory tract, cerebral cortex, striatum, thalamus, hippocampus, hypothalamus, pituitary, midbrain, brainstem, cerebellum. The average SUV of each of the regions was compared between the two groups.

BDNF staining protocol

Sections were stored in 0.01M phosphate-buffered saline (PBS). They were rinsed thrice with PBS and incubated with 3%H2O2 for 20 minutes. The sections were rinsed again with 0.01MPBS and incubated for one hour at room temperature in a solution of 2% normal goat serum and 1% bovine serum albumin (BSA) in 0.01M PBS. Afterwards, the sections were incubated for 3 hours at 37°C with a solution of 1:1000 rabbit-anti-brain-derived neurotrophic factor (BDNF) antibody (Alomone labs, Jerusalem, Israel), 1% BSA, 1% normal goat serum in 0.01M PBS, and then left overnight at room temperature followed by 3 nights in a cold room 4°C. The sections were then washed with 0.01M PBS, and followed by incubation with the secondary 1:500 goat-anti-rabbit antibody (Jackson, Wet Grove, USA) in 0.01MPBS and 1% BSA for one night in the cold room. The sections were rinsed once more in 0.01 M PBS and treated with 1:500 Avidin/Biodin peroxidase Complex (ABC) (Vectastain ABCkit, Vector, Burlingame, USA) vector for two hours at room temperature. The sections were rinsed again and left overnight in the cold room. The staining was completed by adding 3,3’ – daiminobenzidine (DAB) solution of 0.075mg/ml and activated with 0,1% H_2_O_2_. After 24 minutes, the sections were thoroughly washed with 0.01M PBS and left one further night in the cold room. The sections were then mounted and then fixed on glass slides in 1% gelatin followed by dehydration through ethanol and xylol solutions.

## Measured Physiological Parameters

**Table 1** The average (± standard deviation (SD)) heartrate per experimental group before, during and after anaesthesia

|  | Light anaesthesia & normoxia  [/min](Mean ± SD) | Light anaesthesia & hypoxia  [/min](Mean ± SD) | Deep anaesthesia & normoxia  [/min](Mean ± SD) | Deep anaesthesia & hypoxia  [/min](Mean ± SD) |
| --- | --- | --- | --- | --- |
| Before anaesthesia | 315 ± 29 | 340±32 | 34 4± 30 | 347 ± 35 |
| During anaesthesia with or without hypoxia | 353 ± 21 | 346 ± 54 | 345 ± 28 | 383 ± 44 |
| After stop of anaesthesia with propofol | 371 ± 28 | 345 ± 41 | 357 ± 38 | 385 ± 45 |

**Table 2** The average (± standard deviation (SD)) saturation per experimental group before, during and after anaesthesia

|  | Light anaesthesia & normoxia  [%](Mean ± SD) | Light anaesthesia & hypoxia  [%](Mean ± SD) | Deep anaesthesia & normoxia  [%](Mean ± SD) | Deep anaesthesia & hypoxia  [%](Mean ± SD) |
| --- | --- | --- | --- | --- |
| Before anaesthesia | 98 ± 0.8 | 98 ± 2.2 | 99 ± 0.8 | 98 ± 0.8 |
| During anaesthesia with or without hypoxia | 97 ± 1.5 | 97 ± 1.6 | 98 ± 0.9 | 92 ± 7.5 |
| After stop of anaesthesia with propofol | 98 ± 1.0 | 79 ± 6.9 | 98 ± 1.3 | 88 ± 9.7 |

**Table 3** The average (± standard deviation (SD)) systolic blood pressure per experimental group before, during and after anaesthesia

|  | Light anaesthesia & normoxia  [mmHg](Mean ± SD) | Light anaesthesia & hypoxia  [mmHg](Mean ± SD) | Deep anaesthesia & normoxia  [mmHg](Mean ± SD) | Deep anaesthesia & hypoxia  [mmHg](Mean ± SD) |
| --- | --- | --- | --- | --- |
| Before anaesthesia | 87 ± 26 | 85 ± 11 | 90 ± 5 | 88 ± 11 |
| During anaesthesia with or without hypoxia | 124 ± 16 | 124 ± 9 | 114 ± 9 | 119 ± 18 |
| After stop of anaesthesia with propofol | 131 ± 10 | 10 6± 9 | 123 ± 13 | 122 ± 21 |

**Table 4** The average (± standard deviation (SD)) diastolic blood pressure per experimental group before, during and after anaesthesia

|  | Light anaesthesia & normoxia  [mmHg](Mean ± SD) | Light anaesthesia & hypoxia  [mmHg](Mean ± SD) | Deep anaesthesia & normoxia  [mmHg](Mean ± SD) | Deep anaesthesia & hypoxia  [mmHg](Mean ± SD) |
| --- | --- | --- | --- | --- |
| Before anaesthesia | 71 ± 25 | 69 ± 12 | 79 ± 8 | 72 ± 9 |
| During anaesthesia with or without hypoxia | 98 ± 17 | 104 ± 10 | 97 ± 7 | 96 ± 13 |
| After stop of anaesthesia with propofol | 110 ± 7 | 84 ± 9 | 106 ± 12 | 95 ± 20 |

## Photographs for each staining

Below are photographs for the relevant staining’s.


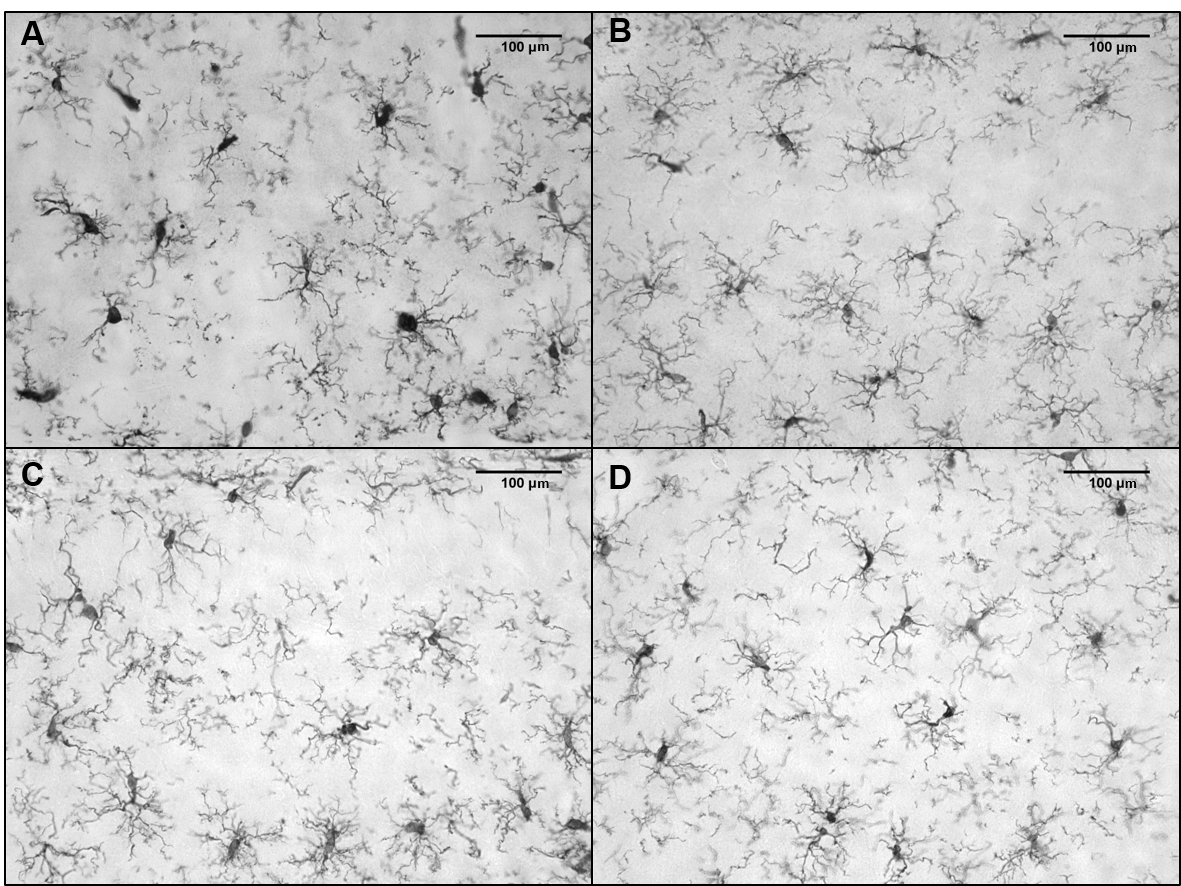


**Figure 1** Detailed photographs (200x magnification) of Iba-1 staining of the hippocampus per group: light-anaesthesia hypoxia (A), deep-anaesthesia hypoxia (B), light-anaesthesia normoxia (C) and deep-anaesthesia normoxia (D). Scale bar illustrated in the top right corner of each figure is 100µm in length.


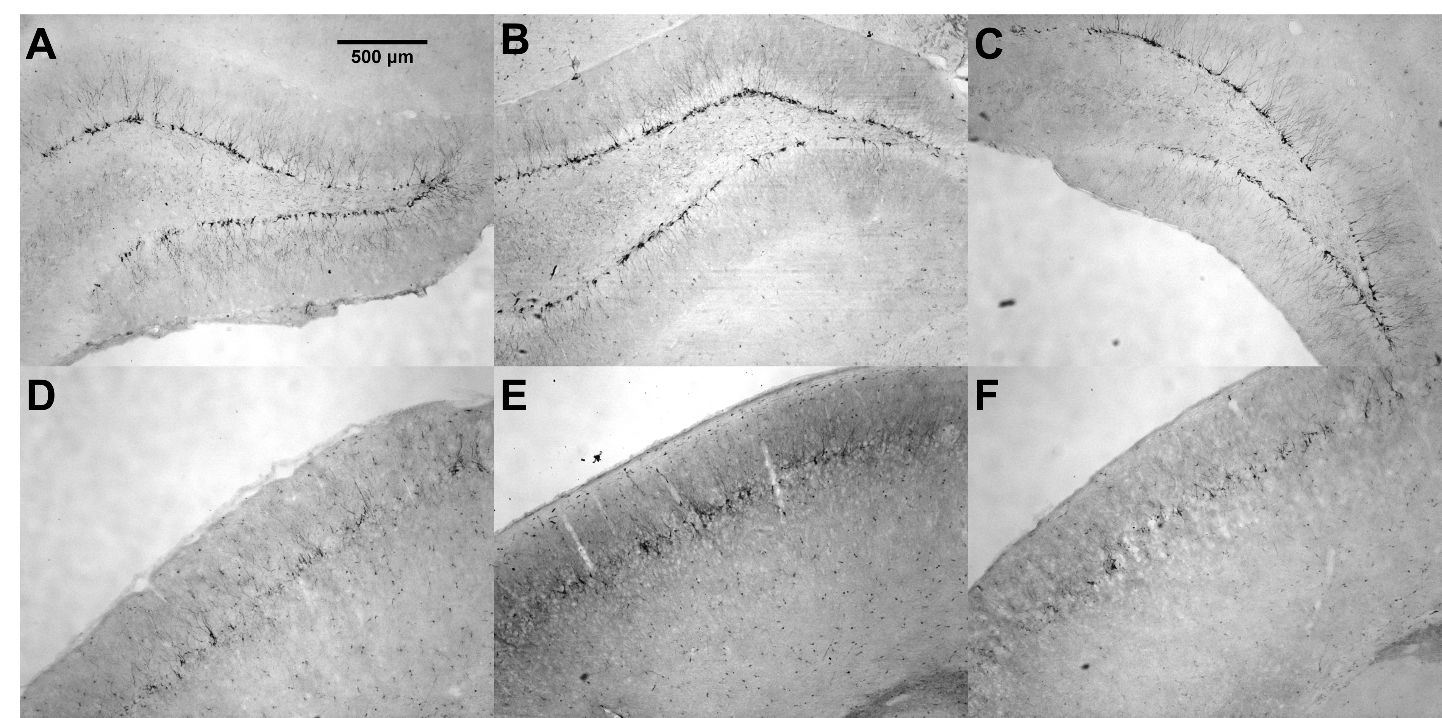


**Figure 2** Overview photographs (50x magnification) of typical DCX staining for the Dentate Gyrus (A, B, C) and Piriform cortex (D, E, F) for the group: control (A, D), light anaesthesia-hypoxia (B, E), and high anaesthesia-hypoxia (C, F). The scale bar (500µm in length) on figure A applies to B, C, D, E and F.


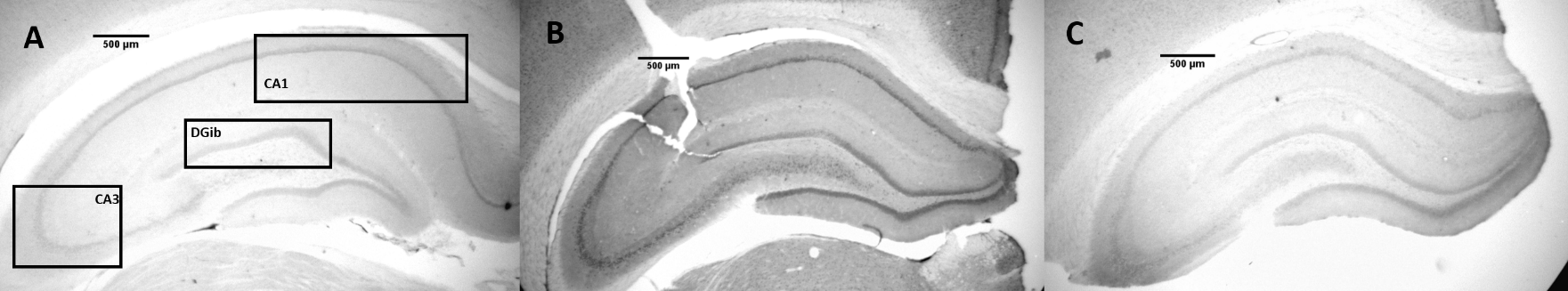


**Figure 3** Overview photographs (12.5x magnification) of typical BDNF staining of the Cornu Ammonis-1(CA1), Dentate Gyrus inner blate (DGib) and Cornu Ammonis-3 (CA3) for the groups: control (A), light anaesthesia-hypoxia (B), and high anaesthesia-hypoxia (C). Scale bar illustrated in the top left corner of each figure is 500µm in length.

## Tables for correlations performed

**Table 5** Positive and negative correlations for the time the rats spent exploring. Iba-1: Ionized calcium-binding adaptor protein-1; CA1: Cornu Ammonis-1; DCX: Doublecortin; BDNF: brain derived neurotrophic factor; DG: Dentate Gyrus inner blate.

|  | | Iba-1 positive cell body size CA1 | Iba-1 positive cell body size DG | DCX in the amygdala | BDNF CA1 | BDNF DG |
| --- | --- | --- | --- | --- | --- | --- |
| Time spent exploring | Pearson correlation | -.350 | -.352 | .477 | -.507 | -.468 |
|  | P= | 0.046 | 0.041 | 0.008 | 0.027 | 0.043 |

**Table 6** Positive and negative correlations for Doublecortin (DCX) expression in the Dentate Gyrus. Iba-1: Ionized calcium-binding adaptor protein-1; CA1: Cornu Ammonis-1.

|  | | Microglia activity in CA1 | Iba-1 positive cell size CA1 | Iba-1 positive dendrite area CA1 |
| --- | --- | --- | --- | --- |
| DCX in the Dentate Gyrus | Pearson correlation | .480 | -.375 | -.382 |
|  | P= | 0.003 | 0.026 | 0.024 |

**Table 7** Positive and negative correlations for brain derived neurotrophic factor (BDNF) expression in Cornu Ammonis-1 (CA1), Cornu Ammonis 3 (CA3) and Dentate Gyrus inner blate (DG). Iba-1: Ionized calcium-binding adaptor protein-1;

|  | | total measured positive Iba-1 area for CA1 | Iba-1 positive cell size CA1 | Iba-1 dendrite area CA1 | total measured positive Iba-1 area for DG |
| --- | --- | --- | --- | --- | --- |
| BDNF CA1 | Pearson correlation | .750 | .598 | .589 | .698 |
|  | P= | 0.000 | 0.005 | 0.006 | 0.000 |
| BDNF CA3 | Pearson correlation | .568 | .529 | .523 | .503 |
|  | P= | 0.014 | 0.024 | 0.026 | 0.028 |
| BDNF DG | Pearson correlation | .685 | .571 | .561 | .657 |

## References

1. Cizek J, Herholz K, Vollmar S, Schrader R, Klein J, Heiss WD. Fast and robust registration of PET and MR images of human brain. Neuroimage. 2004;22: 434-442.
